# Supplementary material for: A panel of diverse Klebsiella pneumoniae clinical isolates for research and development
Source: Microb Genom. 2023 May 4;9(5):mgen000967. doi: 10.1099/mgen.0.000967 (PMC10272860; doi:10.1099/mgen.0.000967)
Supplement: Supplementary material 1 [file mgen-9-967-s001.pdf]

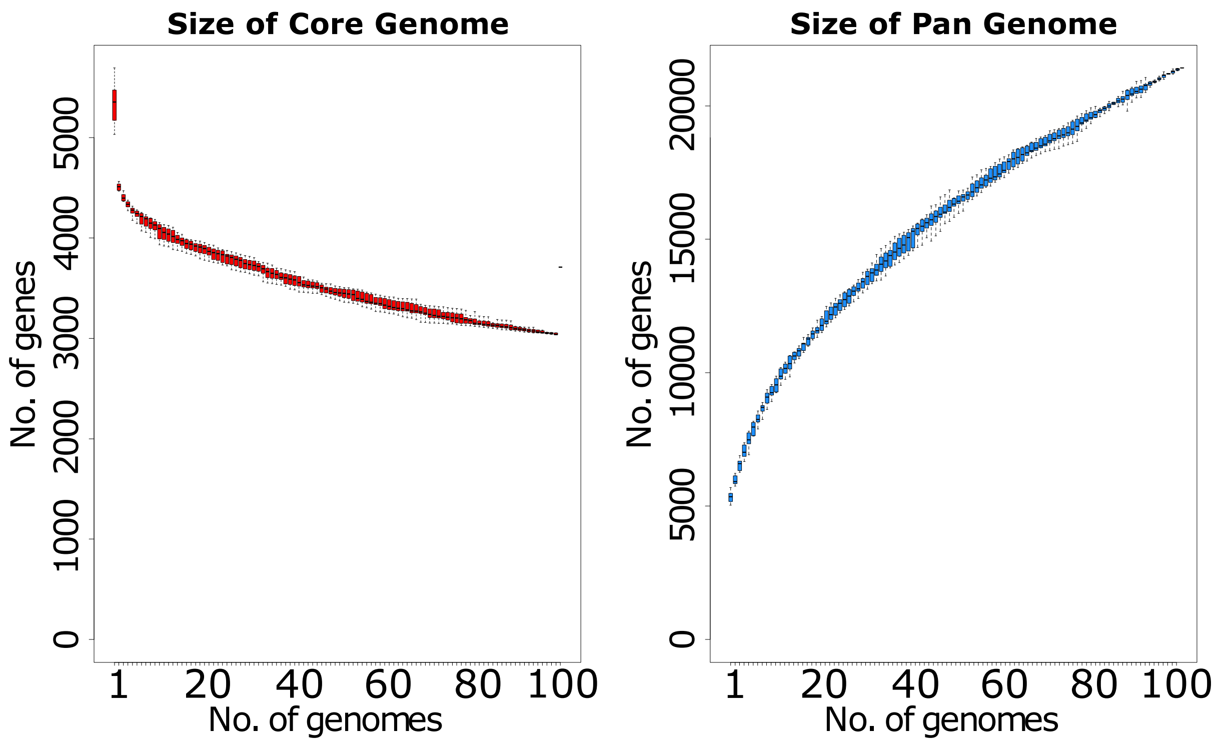

Supplementary Figure 1. Core and pan genome sizes of 100 diverse K. pneumoniae isolates. Gene rarefaction (left, core-genome) and accumulation (right, pan genome) curves are provided with the number of genes (y-axis) as a function of the number of genomes (x-axis). Core genome is defined as the number of genes found in 99% of the genomes. Boxplots indicate the variation in the number of genomes used for the calculation at each position.
